# Nitric oxide releasing coatings for the prevention of viral and bacterial infections†

**DOI:** 10.1039/d4bm00172a

**Published:** 2024-07-09

**Authors:** Jenny Aveyard, Siobhan Richards, Man Li, Graeme Pitt, Grant L. Hughes, Asangaedem Akpan, Riaz Akhtar, Ahmed Kazaili, Raechelle A. D'Sa

**Affiliations:** a School of Engineering, University of Liverpool Harrison Hughes Building Brownlow Hill Liverpool L69 3GH UK r.dsa@liverpool.ac.uk; b Departments of Vector Biology and Tropical Disease Biology, Centre for Neglected Tropical Disease, Liverpool School of Tropical Medicine Pembroke Place Liverpool L3 5QA UK; c Department of Musculoskeletal & Ageing Sciences, University of Liverpool Liverpool L69 3GL UK; d Liverpool University Hospitals NHS FT Liverpool L7 8XP UK; e Department of Biochemistry & Systems Biology, University of Liverpool Liverpool L69 7ZB UK

## Abstract

Healthcare associated infections (HCAI) represent a significant burden worldwide contributing to morbidity and mortality and result in substantial economic consequences equating to billions annually. Although the impacts of HCAI have been felt for many years, the coronavirus pandemic has had a profound effect, escalating rates of HCAI, even with extensive preventative measures such as vaccination, personal protective equipment, and deep cleaning regimes. Therefore, there is an urgent need for new solutions to mitigate this serious health emergency. In this paper, the fabrication of nitric oxide (NO) releasing dual action polymer coatings for use in healthcare applications is described. The coatings are doped with the NO donor *S*-nitroso-*N*-acetylpenicillamine (SNAP) and release high payloads of NO in a sustained manner for in excess of 50 hours. These coatings are extensively characterized in multiple biologically relevant solutions and the antibacterial/antiviral efficacy is studied. For the first time, we assess antibacterial activity in a time course study (1, 2, 4 and 24 h) in both nutrient rich and nutrient poor conditions. Coatings exhibit excellent activity against *Pseudomonas aeruginosa* and methicillin resistant *Staphylococcus aureus* (MRSA), with up to complete reduction observed over 24 hours. Additionally, when tested against SARS-CoV-2, the coatings significantly reduced active virus in as little as 10 minutes. These promising results suggest that these coatings could be a valuable addition to existing preventative measures in the fight against HCAIs.

## Abstract 2

Healthcare associated infections are a significant burden worldwide contributing to morbidity and mortality. We report on the development of nitric oxide releasing polymer coatings that have demonstrated excellent antibacterial/antiviral efficacy.

## Graphical abstract

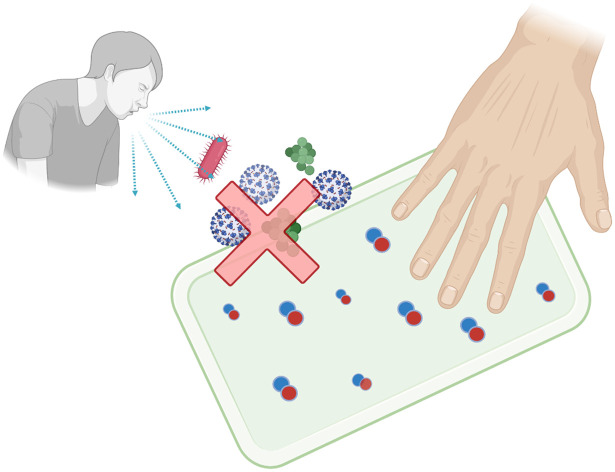

## 1. Introduction

The recent coronavirus pandemic and the increase in the prevalence of antimicrobial resistant organisms has highlighted an urgent need for antibacterial/antiviral strategies especially in healthcare settings. Despite interventions such as personal protective equipment (PPE), diagnostic testing and disinfection of surfaces, the airborne transmission of pathogens and subsequent environmental contamination poses a significant risk to both patients, visitors and healthcare workers. Healthcare-associated infections (HCAI) are a significant cause of morbidity and mortality and represent a substantial economic burden by increasing treatment and length of hospital stay. Additionally HCAI disproportionately affect older adults who are more likely to experience increased severity due to chronic underlying health conditions.^1,2^ Approximately 1 in 25 patients will acquire a HCAI which equates to over $30 billion annually in the US.^3^ Respiratory infections such as influenza and RSV are the most common and account for the highest percentage (22.8%), however this figure has likely increased significantly due to SARS CoV-2.^4^ One recent study reported that as many as 1 in 10 patients admitted to hospital acquired coronavirus disease 2019 (COVID-19) during their stay.^5^ It also reported that this number was significantly higher in residential community care and psychiatric hospitals. Along with the impact of the initial infection, it is also important to consider the far-reaching health and economic consequences of Long COVID, a particular concern in older adults.^6^ A recent study demonstrated that over a third of older adults developed a new health condition in the several months following COVID infection.^2^ Therefore, developing antiviral coatings that can be used widely is urgently required to stem the transmission of viral infection and is aligned with recent guidance from the European Centre of Disease Prevention and Control.^7^

Pathogens associated with HCAI are commonly transmitted in two ways, by an infectious aerosol, defined as the transmission of pathogens through air (*via* droplets) which can occur during natural processes such as breathing, talking, sneezing or coughing or indirectly *via* environmental contamination through transient contact with a surface (fomite).^8^ In terms of aerosol transmission, a recent study in a healthcare facility detected the presence of viable SARS-CoV-2 virus containing particles in a room, over 4 meters away from the infected patients.^9^

Multiple studies have shown that microorganisms such as Methicillin-resistant *Staphylococcus aureus* (MRSA), Vancomycin-resistant *S. aureus* (VRSA), *Clostridium*, *Pseudomonas* and *Enterococcus* and viruses such as SARS-CoV, influenza and norovirus can persist on surfaces for times ranging from hours to days to weeks and even months.^10^ This longevity on the contaminated surfaces can lead to large outbreaks within a community. Indeed, one study showed that up to 40% of HCAI could be attributed to cross contamination from healthcare providers where infection was spread by clothing and gloves or from high touch surfaces such as medical equipment, tables and beds.^11^

The indirect transmission of viruses *via* contaminated surfaces can be the major route for some respiratory viruses.^12^ The SARS-CoV-2 virus has been found to survive on several types of surfaces (plastics, metals, fabrics) for many hours without loss of infectability.^13^ Chia *et al.* found that high touch surfaces were contaminated with viable SARS-CoV-2 in over 70% of COVID patients rooms detectable up to 1 week after admission.^14^ Additionally, a recent study by Chin *et al.* found that infectious SARS-CoV-2 could also be recovered from the outer surfaces of surgical masks after 7 days.^15^ Therefore even in the cases where PPE has been used effectively, there is a high risk of transmission even after the PPE is discarded. Moreover, the disposal of PPE has created extraordinary amounts of plastic waste.^16^

A number of factors can influence the persistence of viruses and bacteria on surfaces including the physical properties of the surface material, for example, roughness, porosity, charge, hydrophobicity/hydrophilicity. The persistence of these pathogens on surfaces can be increased due to stabilisation with proteins or other bacteria present in biological fluid carrier fomites such as sweat, mucus and saliva.

Along with regular cleaning and disinfection, antimicrobial coatings can be a powerful tool in infection control and can help mitigate outbreaks, especially in healthcare settings. The ideal coating should be active against a multitude of pathogens including viruses and bacteria whilst also being non-toxic and environmentally friendly. A recent market report in the US has predicted that the worldwide market for antimicrobial coatings will grow by 10.7% in the next few years, reaching 10 billion by 2025.

Nitric oxide (NO) exhibits broad spectrum antimicrobial activity against a variety of pathogens, including bacteria, viruses,^17^ protozoa and fungi and deleterious effects include damage to viral/bacterial proteins, enzymes and DNA, that are vital to replication and infection of the host. However, NO is a gas with a short half-life, therefore the use of a NO donor is required to enable controlled and sustained delivery and to enable its use in medical devices.^18,19^ The functionalisation of materials with NO donors is well documented and in recent years a plethora of polymer coatings (PVP,^20^ polyurethanes such as Elast-Eon™ and carbosil®, polydimethylsiloxane,)^20–22^ containing *S*-nitrosothiols (RSNOs), such as *S*-nitroso-*N*-acetylpenicillamine (SNAP) and *S*-nitroso-*N*-acetyl-l-cysteine (SNAC) have been produced for medical devices such as urinary^23^ and vascular catheters, cardiovascular stents and bandages.^21,24–26^ These coatings have demonstrated excellent stability and shelf-life, prolonged, localised NO release, and exhibited significant antimicrobial activity, demonstrating their versatility and applicability as antimicrobial coatings for medical applications.^24,27,28^ Handa and coworkers have carried out antimicrobial and antithromobogenic studies both *in vitro* and *in vivo* and have demonstrated the excellent antibacterial properties of polyurethanes such as Elast-Eon containing SNAP.^24,29,30^ This paper extends this work by undertaking a time course antimicrobial evaluation in different nutrient conditions in order to hypothesise the mechanism of this activity. This is the first time such a comprehensive evaluation in various media has been carried out. Furthermore, we have also investigated for the first time the antiviral efficacy of these coatings against two variants of the SARS-CoV-2 virus.

Research has shown that historically viral infection is often accompanied by secondary bacterial infection which results in devastating effects on patient morbidity and mortality. In a review by Gupta *et al.*, studies showed that secondary infection with *Staphylococcus aureus* and *Streptococcus pneumoniae* exacerbated influenza infections, with co-infection leading to 25% of deaths.^31^ Similarly, a study by Gill *et al.* found that during the swine influenza pandemic (H1N1) in 2009, over 55% of patients had a secondary bacterial pneumonia infection caused by *Streptococcus* or *Staphylococcus aureus* which led to an increase in mortality.^32^ In the recent SARS-CoV-2 pandemic, more patients infected with C19 had secondary bacterial infections than influenza patients, and these infections were independently associated with death. Given the prevalence of co-infections, persistence on surfaces, spread of infection from devices, the fabrication of coatings that can demonstrate both antibacterial and antiviral activity for use in medical applications is of vital importance. Furthermore, as SNAP-Elast-Eon is a versatile polymer that can be spin coated or dip coated to form homogeneous layers on many different materials and has been shown to be stable during long term implantation, it could also be applied to medical devices such as endotracheal tubes and nasal cannulas to prevent viral and bacterial colonisation that can lead to ventilator-associated pneumonia.

In this work, we investigate both the antiviral and antibacterial efficacy of these coatings against two strains of SARS-CoV-2 virus (Delta and Omicron) two microorganisms commonly associated with HCAI (MRSA and *Pseudomonas aeruginosa* PAO1)^3^ The stability and NO payload in various conditions is also assessed to determine the feasibility of using polyurethane containing SNAP coatings for medical applications where broad spectrum antibacterial and antiviral activity is required.

## 2. Materials and methods

Sodium nitrite, Luria Bertani (LB) agar and broth, fetal bovine serum (FBS), penicillin, streptomycin and gentamycin were from Merck KGaA, Darmstadt, Germany. (3-(4,5-Dimethylthiazol-2-yl)-2,5-diphenyltetrazolium bromide) (MTT) and formalin were from – VWR. Dulbecco's modified Eagle's medium (DMEM) and Crystal violet were from SLS. *N*-Acetyl-d-penicillamine (NAP) was from Carbosynth, UK. All concentrated acids, solvents and salts were reagent grade and also purchased from Merck KGaA, Darmstadt, Germany. PBS 15 mM sodium phosphate, 0.15 M sodium chloride, pH 7.4. Elast-Eon (E2As) samples were kindly provided by Biomerics (Salt Lake City, UT 84116). Nitrogen, oxygen, argon, nitric oxide calibration (87 ppm in nitrogen), and pure nitric oxide (99%) were purchased from BOC Gases Ltd, Guildford, UK. 2.

Methicillin resistant *Staphylococcus aureus* (MRSA; NCTC 13142) *Pseudomonas aeruginosa* (PAO1;) and Vero C1008 [Vero 76, clone E6, Vero E6] (ECACC 85020206) were purchased from Public Health England, The SARS-CoV-2 Delta variant was passage 5 of a clinical isolate (SARS-CoV-2/human/GBR/Liv_273/2021, GenBank accession OK392641). The SARS-CoV-2 Omicron variant was passage 4 of a clinically isolated strain (SARS-CoV-2/human/GBR/Liv_1326/2021, GenBank accession OP630952).

### 2.1 Synthesis of *S*-nitroso-*N*-acetylpenicillamine (SNAP)

SNAP was synthesised using the method reported by Meyerhoff *et al.*^33^ Briefly, 3 g of NAP was added to a stirred solution containing 75 mL of 1.0 M HCl and 3 mL of conc. H_2_SO_4_. The solution was stirred for 10 minutes and covered with aluminum foil to protect it from light before 15 mL of sodium nitrite solution (2.3 g in 15 mL of distilled H_2_0) was added in 1 mL aliquots over a period of 15 minutes. The solution was stirred at room temperature for 45 minutes before chilling on ice for 10 minutes. The dark green precipitate containing SNAP crystals was then washed to remove unreacted reagents by vacuum filtration with 50 mL of ice cold distilled H_2_O and then the SNAP crystals were dried overnight under vacuum, protected from light. After drying, SNAP was stored in a dark container in the freezer until use.

### 2.2 Preparation of SNAP coated substrates

180 mg of E2As (Structure in ESI Fig. S1†) was dissolved in 3 mL of dry THF by stirring overnight at room temperature. To prepare SNAP coated substrates, 20 or 40 mg of synthesised SNAP was added to 1 mL of E2As solution to prepare 2 and 4% w/v coatings. 50 μl of SNAP-E2As solutions were drop coated onto ethanol cleaned coverslips (19 mm ∅). Surfaces were cured and excess solvent was evaporated under vacuum at room temperature overnight. Coated substrates were protected from light at all times. To prepare substrates with an overcoat, 50 μl of E2As (as prepared above) without SNAP was spin coated using a SCS 6800 spin coater (Speciality coating systems, Indianapolis, IN 46278, USA) onto 4 w/v% SNAP coated substrates at 5000 rpm. Overcoat substrates were also cured and excess solvent removed at room temperature under vacuum overnight (protected from light).

### 2.3. Atomic force microscopy analysis

Atomic force microscopy (AFM) was used to observe changes in surface topography. The samples were scanned using an AFM Multimode 8 (Bruker, Billerica, MA, USA) system fitted with a NanoScope V controller and a J-Scanner. A silicon probe (RTESPA-150, Bruker, CA, USA) with a nominal radius of 8 nm was used for scanning the samples in ambient conditions. ScanAsyst mode in air was utilised at a scan rate of 0.606 Hz, at resolution of 512 indent/line and scan size of 50 μm to capture the topography of the samples. NanoScope Analysis v1.9 software (Bruker, Billerica, MA, USA) was used to obtain roughness data. Root mean square roughness (*R*_q_) and average roughness (*R*_a_) were calculated from at least three replicates of each sample type, from at least three points per sample. All data are presented as mean average ± standard deviation.

### 2.4 Chemiluminescence measurement of NO release

Nitric oxide release from SNAP coated substrates was determined using a Sievers 280i Chemiluminescence NO analyser (NOA; Boulder, CO). Each sample was analysed in triplicate in LB broth, cell culture media (DMEM) and PBS to determine NO release measurements under all experimental conditions. DMEM was used as received and not supplemented with additional serum protein. To investigate the mechanism of NO release in more detail, 4 w/v% SNAP coated substrates were also measured in triplicate in LB broth, DMEM and PBS containing 100 μM EDTA. These measurements were obtained at room temperature in both light and dark conditions. Prior to sample measurement, the NOA was calibrated according to the manufacturer's protocol using NO standard gas (87 ppm) and air passed through a NO zero filter (0 ppm of NO). To measure NO release from the samples, SNAP coated substrates were submerged in 5 mL of measurement solution that was purged with nitrogen gas at a flow rate of 70 mL min^−1^ to carry released NO from the solution to the NOA. Additional nitrogen flow was supplied to the flask to match the collection rate of the instrument (200 mL min^−1^).

### 2.5 Leaching study

SNAP coated substrates were soaked in 5 mL of PBS, pH 7.4, containing 100 μM EDTA in the dark at room temperature. To determine the concentration of SNAP released from the substrates, aliquots of the solution were removed at time points corresponding to the antimicrobial and virological experiments. Aliquots were protected from light and analysed by UV–Vis spectrometry. PBS buffer containing control coated substrates were used as the blank. The molar absorption coefficient for SNAP in PBS at 340 nm was determined to be: *ε*_SNAP_ = 1024 m^−1^ cm^−1^.^24^ The % SNAP leached from the substrate was determined by the difference between the initial concentration of SNAP in the substrate minus the amount of SNAP that had leached into the PBS. All measurements were obtained in triplicate.

### 2.6 pH measurements

To investigate the pH of solutions upon NO release, SNAP coated substrates were submerged in 2 mL aliquots of distilled H_2_0, PBS, DMEM or LB broth either with or without EDTA. Measurements were obtained after 1, 2, 4 and 24 hours incubation at both room temperature and 37 °C in triplicate using a Jenway 3510 pH meter.

### 2.7 Antimicrobial efficacy of SNAP coatings

The antimicrobial efficacy of the SNAP coated substrates was observed as a reduction in colony forming units (CFU) of MRSA or PAO1 using a bactericidal assay in either nutrient rich (LB broth) or nutrient poor (PBS) conditions. For nutrient poor assays, overnight cultures of bacteria were centrifugally precipitated at 5000 rpm for 5 minutes to remove LB broth, and bacterial pellets were washed with PBS twice before resuspension in 1 mL of PBS. For nutrient rich assays, overnight cultures remained in LB broth. Cultures were then diluted to 1 × 10 ^6^ CFU mL^−1^ (by determination of absorbance at 600 nm compared to a 0.5 McFarland Reference Standard). Coated substrates (19 mm ∅) were incubated with 1 mL of the bacterial solution at 37 °C for 1, 2, 4 and 24 hours respectively. At the end of this time, a serial dilution (in either LB for nutrient rich or PBS for nutrient poor conditions) was performed on LB agar using the Miles and Misra method. Biofilm assays in nutrient rich and nutrient poor conditions were also performed to determine if SNAP coated substrates resisted the adhesion of biofilms. In these assays substrates were incubated with 1 mL of MRSA or PAO1 (1 × 10 ^6^ CFU mL^−1^) in either LB broth or PBS at 37 °C for 24 hours. At the end of this time, substrates were removed to fresh wells and planktonic bacteria was removed by gently washing in PBS three times. Adhered biofilm bacteria was then removed from the substrates by mechanical vibration in PBS. This solution was then serially diluted in either LB or PBS and plated on LB agar using the Miles and Misra method. All substrates were studied in triplicate and repeated three times. Statistical analysis was performed using the data analysis package SigmaPlot 13.0 (Systat Software, San Jose, CA). One-way analysis of variance (ANOVA) was used to establish variance between SNAP coated substrates and *p* values < 0.05 were determined to be statistically significant.

### 2.8 Virology assays

All SARS-CoV-2 work was conducted in a level 3 containment laboratory. For virucidal efficacy assays, Vero E6 cells were maintained in DMEM with 10% foetal bovine serum (FBS) and 0.05 mg mL^−1^ gentamicin, at 37 °C with 5% CO_2_. Virus was cultured in DMEM with 2% FBS and 0.05 mg mL^−1^ gentamicin, at 37 °C with 5% CO_2_. After 72 hours virus was harvested and stored at −80 °C for later use. The virucidal efficacy of the SNAP coated substrates was observed as a reduction in plaque forming units (PFU) of either SARS-CoV-2 Delta or Omicron variant using a virucidal activity assay. SNAP coated or polymer control substrates were incubated with 1 mL of SARS-CoV-2 for 10 min, 30 min, 2 or 4 hours. At the end of this time, remaining virus was resuspended and serially diluted to determine viral titre using a plaque assay. For the plaque assay, cells were incubated at 37 °C with 5% CO_2_ for 72 hours, after which they were fixed with formalin, and stained with 0.25% crystal violet solution. All samples were studied in triplicate and repeated three times. Statistical analysis was performed using the data analysis package SigmaPlot 13.0 (Systat Software, San Jose, CA). One-way analysis of variance (ANOVA) was used to establish variance between SNAP coated substrates and *p* values < 0.05 were determined to be statistically significant.

### 2.9 Cytocompatibility

Leachates from control samples and SNAP samples were obtained by soaking the samples (*n* = 3) in DMEM medium (1 mL of medium per sample) and incubated for 4 and 24 h time points at 37 °C with 5% CO_2_ supply.

Mouse fibroblast cells (NCTC L929) were cultured on 75 cm^2^ T-flasks in Dulbecco's modified Eagle's medium (DMEM) containing 1 g L^−1^ glucose, 10% FBS, and 1% penicillin–streptomycin at 37 °C under a humidified atmosphere with 5% CO_2_. Once confluency reached 90%, the cells were trypsinised and seeded in 96-well plates with a concentration of 1 × 10^5^ cells per well at 37 °C, 5% CO_2_. The 96-well plate was then incubated for 24 h, after which growth media were removed from the wells and 100 μL leachate media from materials, controls and plain culture medium were added to the cells. The cells were incubated for a further 24 hours. Subsequently, all liquids were removed from wells and 100 μL of sterilised PBS were added in the well to wash off the media residuals. After PBS was removed from each well, 100 μL of 1 mg mL^−1^ MTT solution was added for a further 4 h incubation at 37 °C, 5% CO_2_, following with the addition of 100 μL DMSO solution into each well for 10 min at room temperature in the dark to complete solubilisation of the purple formazan crystals. The absorbance of the samples was measured using a plate reader at OD = 570 nm. All substrates were studied in triplicate and repeated three times. Data taken were expressed as mean ± standard deviation. Statistical analysis was carried out using a Tukey's *t*-test with SigmaPlot software. *P* value <0.05 was considered statistically significant for all data throughout the study.

## 3. Results and discussion

### 3.1 Surface topography: AFM

**Fig. 1:**
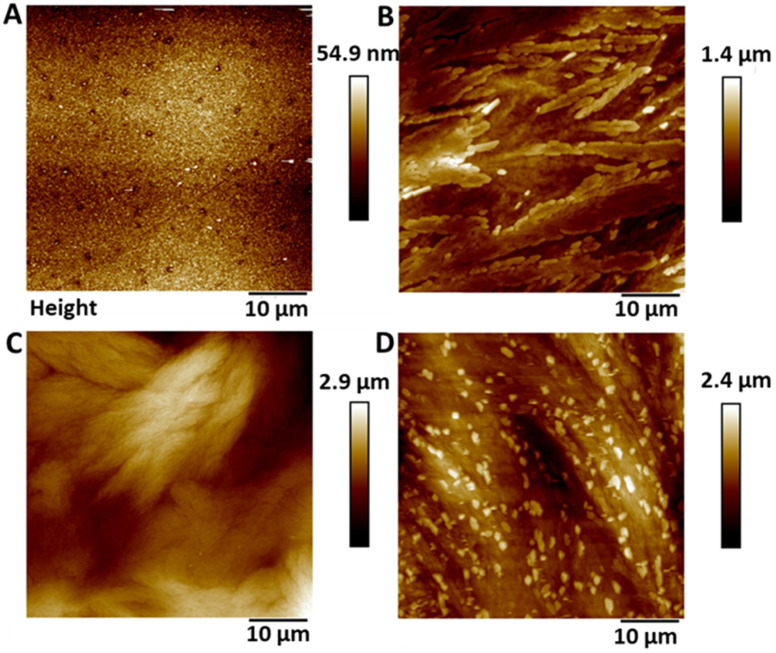
Representative AFM height profiles of (A) Control Elast-Eon (B) 2 w/v% (C) 4 w/v% and (D) 4 w/v% OC. As the concentration of SNAP in the coating is increased, the surface height/topography increases.

Atomic force microscopy (AFM) was used to determine the surface topography of the SNAP coated substrates and representative height profiles are displayed in Fig. 1. AFM derived data including surface features and roughness values (*R*_q_, *R*_a_) are presented in Table 1 and graph with data can be seen in ESI Fig. S2.† The control surfaces were relatively smooth with no features and a *R*_a_ value of 5.37 nm ± 1.08. The surface roughness values increased with increasing concentrations of SNAP incorporated into the Elast-Eon polymer. The addition of an overcoat to the 4 w/v% sample did not statistically significantly change the topography of the surface indicating a very thin polymer overcoat was present.

**Table 1:** AFM derived roughness values (*R*_q_/*R*_a_) for SNAP coated substrates. Values represent the mean value obtained from three measurements on three separate areas of the substrate

Control		2 w/v%		4 w/v%		4 w/v% OC	
*R*_q_	*R*_a_	*R*_q_	*R*_a_	*R*_q_	*R*_a_	*R*_q_	*R*_a_
6.87 ± 1.42	5.37 ± 1.08	236.6 ± 25.4	189.3 ± 22.1	442.3 ± 107	341.3 ± 82.1	467.6 ± 99.4	378.6 ± 89.6

### 3.2 Chemiluminescent Detection of NO in different media

**Fig. 2:**
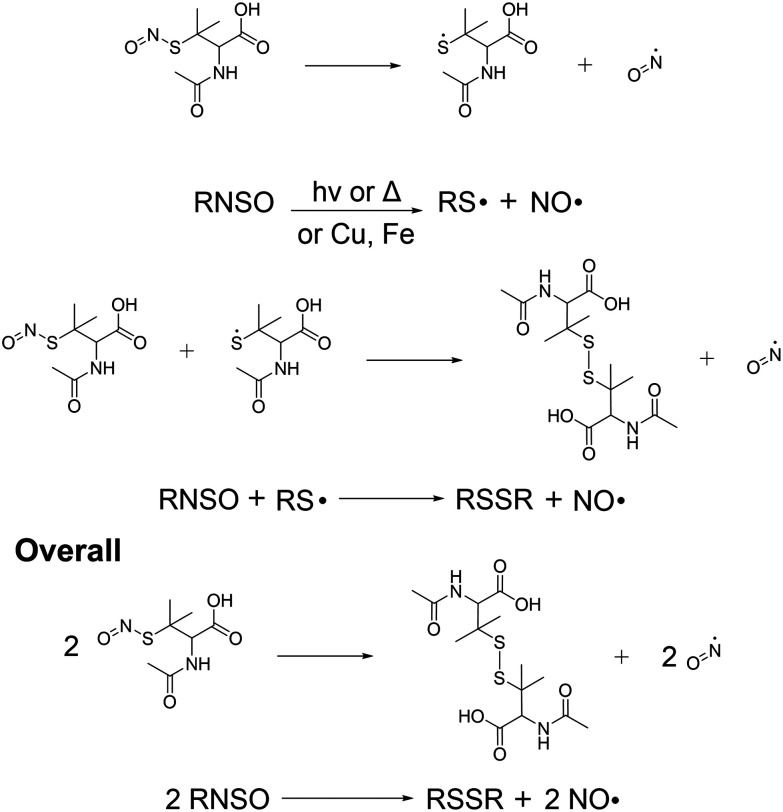
Reaction scheme detailing the release of NO from SNAP coated substrates. The release of NO can be catalysed by light, thiols, heat or metal ions.

Release of NO from the SNAP-coated substrates was detected in real time using a chemiluminescence nitric oxide analyser. In recent years the RSNO NO donor SNAP has been incorporated into a variety of materials including Elast-Eon, where it has been observed to exhibit remarkable stability and significant broad-spectrum antimicrobial activity making it an ideal coating for use in medical applications. SNAP decomposes to produce NO and a thiyl radical when the S–N bond is homolytically cleaved by light, heat, thiols and metal ions such as Cu^2+^ and Fe^2+^ (Fig. 2). If the concentration of SNAP is high, the thiyl radical can further react with SNAP to produce more NO. SNAP decomposition and subsequent NO release is highly dependent on the chemical environment therefore when determining the payload of NO released from SNAP functionalised materials, it is important to consider different release media. Many studies have shown that different pH's, buffer components and concentrations can affect the rate and concentration of NO released from SNAP and specifically NO payload from functionalised materials can be affected by complex solutions that contain additives such as amino acids and vitamins that can scavenge/quench NO.^34–36^

We performed chemiluminescence measurements in three different solutions: PBS (pH 7.6), DMEM (pH 8) and LB broth (pH 7.1) (Table 2) to accurately determine the payload of NO released from SNAP coated substrates in the solutions used for bacterial, cytocompatibility and virology assays. The majority of samples released NO in excess of >40 hours, however to allow for fair comparison between samples and solutions, we calculated NO payloads in the first 24 hours of release.^37^ We observed significant differences in the NO payload depending on the measurement solution. The highest payloads of NO were observed in PBS solution (Table 1). Substrates coated with the lowest concentration of SNAP (2 w/v%) released 3.08 ± 0.9 × 10^−10^ mol cm^−2^ min^−1^ over the 24-hour period. When the concentration of SNAP was increased to 4 w/v% we observed 77% increase to 13.7 ± 0.7 × 10^−10^ mol cm^−2^ min^−1^. We also investigated whether the inclusion of an additional hydrophobic polymer overcoat layer to the 4 w/v% substrate could affect the concentration and longevity of NO release. We observed a 63% decrease in NO payload (5.0 ± 2 × 10^−10^ mol cm^−2^ min^−1^) compared to the 4 w/v% substrate. This is in agreement with numerous studies by Handa *et al.* who observed that the addition of a hydrophobic overcoat limits water uptake to SNAP coated substrates, which in turn reduces leaching and slows subsequent NO release.^29^

**Table 2:** Amounts of NO released from substrates coated with 2 w/v%, 4 w/v% and 4 w/v% SNAP with overcoat in different assay solutions (LB, DMEM and PBS, protected from light at room temperature (*N* = 3))

Sample	[NO]_max_ (×10^−10^ mol cm^−2^ min^−1^)	Average *t*_max_ (min)	[NO] 4 h (×10^−10^ mol cm^−2^ min^−1^)	[NO] 24 h (×10^−10^ mol cm^−2^ min^−1^)	*t*[NO]4 h mol cm^−2^	*t*[NO]24 h mol cm^−2^
LB 2 w/v%	0.84 ± 0.7	9.3	0.25 ± 0.7	0.25 ± 0.2	6 × 10^−9^	35 × 10^−9^
LB 4 w/v%	2.6 ± 1	23	0.8 ± 0.37	0.59 ± 0.51	19.2 × 10^−9^	82 × 10^−9^
LB OC	1.2 ± 0.24	7.7	0.42 ± 0.32	0.36 ± 0.06	10.1 × 10^−9^	50 × 10^−9^
DMEM 2%	8.8 ± 3.8	78	4.3 ± 0.4	1.2 ± 0.4	103 × 10^−9^	168 × 10^−9^
DMEM 4%	14.6 ± 0.79	55	5.8 ± 0.61	4.2 ± 1.5	139 × 10^−9^	588 × 10^−9^
DMEM OC	8.2 ± 0.42	34	2.3 ± 1.2	1.86 ± 0.4	55 × 10^−9^	260 × 10^−9^
PBS 2%	7.5 ± 0.27	18	3.3 ± 1.1	3.08 ± 0.9	79 × 10^−9^	420 × 10^−9^
PBS 4%	18.4 ± 0.05	29	6.2 ± 0.6	13.7 ± 0.7	149 × 10^−9^	1.92 × 10^−6^
PBS OC	9.3 ± 0.98	10	6.3 ± 0.29	5.0 ± 2	151 × 10^−9^	700 × 10^−9^

In DMEM cell culture media all substrates had a lower NO payload than that measured in PBS. The 2 w/v% substrate released 1.2 ± 0.4 × 10^−10^ mol cm^−2^ min^−1^ over the 24-hour period, the 4 w/v% released 4.2 ± 1.5 × 10^−10^ mol cm^−2^ min^−1^, whilst the overcoated substrates released 1.86 ± 0.4 × 10^−10^ mol cm^−2^ min^−1^ (Table 1). This corresponded to 61%, 69% and 62% reduction when compared to PBS measurements. A number of factors are responsible for the decrease in NO payload we observed, for example, it has been reported that the presence of additives in complex solutions can result in lower concentrations of NO due to scavenging. Harding *et al.* conducted a comprehensive study comparing the release of NO from three different NO donors in different cell culture media and solutions and observed a significant decrease in the NO measured in neurobasal A media compared to PBS.^34^ They attributed this to the presence of a combination of tryptophan and vitamin B2 (riboflavin) and the extent of NO scavenging could be as high as 70% depending on the tryptophan/riboflavin ratio. The depletion of NO was also dependant on the half-life of the NO donor and the concentration. Our coatings were measured in DMEM that contains both tryptophan and riboflavin, which will account for some of the reduction in measured NO. Additionally, DMEM also contains a high concentration of glucose (20 mM). Goligorsky *et al.* studied the effects of increasing glucose concentration on NO production by endothelial cells and found that it was 10–20% lower in the presence of 20 mM glucose. They also confirmed that this effect was not only seen in a biological scenario by spiking NO gas saturated solutions with glucose. Although they were unable to confirm the mechanism of this reaction, they attributed this decrease to the scavenging of NO by the glucose.^38^ Our results confirm that NO is scavenged in DMEM and also confirm that the addition of the hydrophobic E2As overcoat both slows the leaching of the NO from the substrate and acts as a protective barrier against scavengers.

When the same substrates were measured in LB broth the concentration of NO released was significantly lower. The amount of NO released from the substrate coated with 2 w/v% SNAP was 0.25 ± 0.2 × 10^−10^ mol cm^−2^ min^−1^ over 24 hours. The amount of NO released from 4 w/v% and 4 w/v% with an overcoat were 0.59 ± 0.51 and 0.36 ± 0.06 × 10^−10^ mol cm^−2^ min^−1^ respectively. The amount of NO released from the 4 w/v% sample was over 95% lower in LB broth than in PBS, and 86% lower than in DMEM. Similarly, to DMEM, LB broth is comprised of additives that can scavenge NO. LB contains tryptone and yeast extract which contain both amino acids and B vitamins. Vitamins B6 and B12 have previously been shown to scavenge NO^39^ and LB also contains riboflavin (vitamin B2) in combination with tryptophan which could explain the lower payloads of NO measured in this solution.

### 3.3 Leaching study

**Fig. 3:**
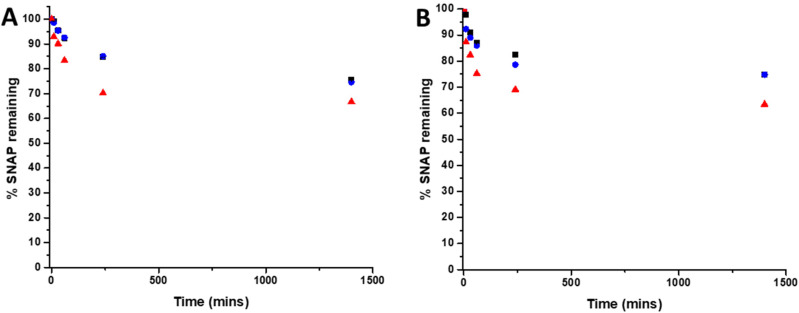
Graphs showing % SNAP remaining in SNAP coated substrates at (A) room temperature (RT) and (B) 37 °C. Black 2 w/v%; blue 4 w/v%/OC; red 4 w/v%. Values represent mean ± SD (*n* = 3).

To determine the amount of SNAP leaching from coatings over time, substrates were soaked in PBS solution (containing EDTA), at room temperature and at 37 °C. At time points corresponding to those of the biological experiments, aliquots of solution were removed and analysed by UV Vis spectroscopy. Fig. 3 shows the % SNAP remaining in the substrates after soaking. As SNAP concentration in the substrates increases from 2 w/v% to 4 w/v%, the % of SNAP leached from the substrates increases. Results also demonstrate that the addition of the overcoat of E2As slows down the release of NO. At room temperature, when substrates are initially soaked in PBS, we observed an increase in absorbance at 340 nm within the first hour, suggesting a burst release of SNAP as the E2As polymer absorbs water. This corresponded to the loss of 16.6% SNAP from the 4 w/v% substrate and 7% loss from both the 2 w/v% and 4 w/v% OC substrates respectively. SNAP continued to leach from the substrates and a 16% loss from 2 w/v%, 15% loss from 4 w/v%OC and 30% loss from 4 w/v% was observed at the 4-hour time point. After 24 hours of soaking, the amount of SNAP leached from the substrates was 25% from the 2 w/v% and 4 w/v% OC and 33% from the 4 w/v% substrate indicating that the amount of SNAP leaching from substrates was beginning to slow. The leaching of SNAP from the coated substrates is slightly faster at elevated temperatures (room temperature *vs.* 37 °C), particularly at the earlier time points (between 10 min–1 h). From 1 hour onwards, the leaching at 37 °C slows to match that of room temperature. This effect is due to the higher temperature (37 °C) allowing for an increased water uptake into the polymer. These results are in agreement with numerous studies by Handa and coworkers and demonstrate that E2As would be a suitable polymer coating for long-term release of NO/SNAP.^24,29^

### 3.4 pH studies

**Fig. 4:**
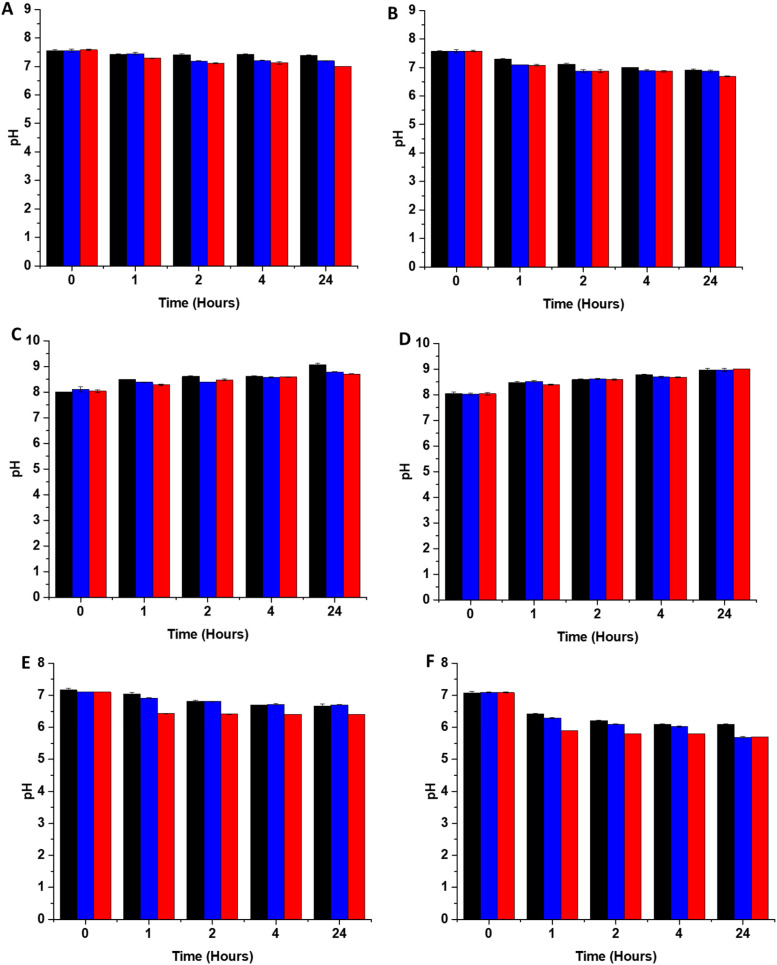
pH changes of solutions containing SNAP coated substrates at room temperature (RT) and at 37 °C (A) PBS RT (B) PBS 37 °C (C) DMEM RT (D) DMEM 37 °C (E) LB broth RT (F) LB broth 37 °C. Black 2 w/v%; blue 4 w/v%/OC; red 4 w/v%. The pH change observed upon release of HNO_2_ is dependent on the amount of immobilised SNAP and the buffering capacity of the solution. Values represent mean ± SD (*n* = 3).

In addition to scavenging/quenching agents in complex solutions, studies have shown that the pH of the solution can also have a significant effect on the stabilisation/destabilisation of SNAP and NO release/payloads. Additionally, pH has also been shown to affect the production of reactive species such as peroxynitrite from NO, which in turn may affect antimicrobial/antiviral efficacy. As a result, it is important to consider the effects of pH in the design of coatings for medical applications. In unbuffered solutions, any generated NO is converted to mildly acidic HNO_2_. Studies have shown that SNAP (and other RSNOs) are more stable in acidic conditions due to protonation of the oxygen on the RSNO which in turn increases the strength of the S–N bond. This reduces decomposition and subsequently release of NO is slowed. If the solution is buffered at physiological pH, the pH of the solution remains stable upon release of NO, which results in further SNAP decomposition and NO release as it is less stable at this pH. In a study by Reynolds *et al.*, SNAP exhibited the highest stability at pH 5, and was least stable at pH 7.4. They also observed a decreased stability when the pH of the solution was very acidic (pH 3).^35^ In another study, Wang *et al.* found that the lifetime of SNAP varied from 0.5 to 4 days dependent on the buffer components, pH and concentration.^36^ In order to determine the stability of SNAP substrates used in this work, we measured the pH of 3 different solutions, DMEM, LB broth and PBS after 1, 2, 4- and 24-hours incubation with the substrates at both room temperature and 37 degrees. From Fig. 4, it can be seen that the pH change due to release of HNO_2_ differs in PBS, DMEM and LB broth this is a result of the different buffering capacity of these solutions.

#### PBS

At room temperature, in PBS (Fig. 4A), which has the highest buffering capacity, the pH of the solution containing the 4 w/v% substrate decreases from 7.6 to 7.0 over a period of 4 hours and then remains at this pH for the 24-hour measurement period. In PBS containing the 2 w/v% and 4 w/v% overcoated substrates, we also observe the same trends although the pH changes occur more slowly as the concentration of SNAP, and subsequent HNO_2_ produced is lower in these samples. At 37 °C in PBS (Fig. 4B), the pH of the solution containing the 4 w/v% substrate decreases slowly from 7.6 to 6.9 over a period of 4 hours and then decreases further to 6.7 after 24-hours. In the solution containing the 2 w/v% substrates the pH 7.6 to 7 over 4 hours then to 6.9 over the 24-hour period. For, the 4 w/v%, the pH decreased from 7.6 to 6.9 within 4 hours and the pH was maintained at 6.9 over the 24-hour period.

#### DMEM

At room temperature, in DMEM the pH increases over the 24-hour period for all samples measured (Fig. 4C). This is due to the presence of bicarbonate in the DMEM solution and the absence of CO_2_, which would usually maintain a physiological pH in cell culture experiments. In DMEM at 37 °C (Fig. 4D) the pH of all solutions again increases to 9 over the 24-hour period due to the absence of CO_2_.

#### LB broth

At room temperature in LB broth the pH of the solution containing the 4 w/v% substrate decreases from 7.1 to 6.4 in 4 hours and is then maintained at this pH for the 24-hour measurement period (Fig. 4E). Both LB broth solutions containing the 2 w/v% and the 4 w/v% OC substrates decrease from a pH of 7.1 to 6.7 more slowly over the 24-hour measurement period. At 37 degrees, in broth the pH of the solution drops considerably for all substrates (Fig. 4F). In the solution containing the 4 w/v% substrate the pH drops from 7.1 to 5.8 in the first 4 hours then decreases further to 5.6 over the 24 hours. The pH of the 2 w/v% solution decreases from 7.1 to 6.1 in the first 4 hours then remains at 6.1 for the 24-hour measurement period. The 4 w/v% with overcoat pH decreases from 7.1 to 6 within the 4 hours, then decreases further to 5.7 over 24 hours.

The pH of all substrates in all solutions tested decreases faster and is lower at 37 °C due to an overall increase in NO payload released from substrates at higher temperatures which will result in more acidic pHs. Brisbois *et al.*, studied NO release from SNAP coated medical polymers in different conditions and determined that NO payloads were over 30% higher at 37 °C compared to room temperature.^24^

### 3.5 Mechanism of release: metal ions *vs*. light

Previous studies have shown that low copper ion concentrations (10^−5^ to 10^−6^ M) such as those present in buffers are capable of decomposing SNAP. In order to study the release mechanism of NO from SNAP coated substrates in more detail we measured the NO payloads released from 4 w/v% samples in DMEM, LB broth and PBS with and without EDTA, in the presence or absence of light (Table 3).

**Table 3:** Total NO released for 4 w/v% substrates in LB, DMEM and PBS solutions with and without EDTA at room temperature in the absence of light (*N* = 3)

Sample	[NO]_max_ (×10^−10^ mol cm^−2^ min^−1^)	Average *t*_max_ (min)	[NO]_4 h_ (×10^−10^ mol cm^−2^ min^−1^)	[NO]_24 h_ (×10^−10^ mol cm^−2^ min^−1^)	*t*[NO]4 h mol cm^−2^	*t*[NO]24 h mol cm^−2^
LB/dark	2.6 ± 1	23	0.8 ± 0.37	0.59 ± 0.51	19.2 × 10^−9^	826 × 10^−9^
LB/dark EDTA	1.53 ± 0.4	3.9	2 ± 1	0.1 ± 0.05	48 × 10^−9^	140 × 10^−9^
LB/light	34.3 ± 1	112	26 ± 1	7.5 ± 0.03	624 × 10^−9^	1.05 × 10^−6^
LB/light EDTA	44 ± 0.72	91	32 ± 0.4	3.9 ± 2	768 × 10^−9^	546 × 10^−9^
DMEM/dark	14.6 ± 0.79	55	5.8 ± 0.61	4.2 ± 1.5	139 × 10^−9^	588 × 10^−9^
DMEM/dark EDTA	2.9 ± 3	119	5.3 ± 0.49	3.4 ± 2	127 × 10^−9^	476 × 10^−9^
DMEM/light	63 ± 4	33	44 ± 3	5.3 ± 0.46	1.0 × 10^−6^	742 × 10^−9^
DMEM/light EDTA	11.6 ± 0.9	93	4 ± 0.5	5.8 ± 0.37	96 × 10^−9^	812 × 10^−9^
PBS/dark	18.4 ± 0.05	29	6.2 ± 0.6	13.7 ± 0.7	149 × 10^−9^	1.92 × 10^−6^
PBS/dark EDTA	6.13 ± 1.5	11	1.5 ± 1	1 ± 0.32	36 × 10^−9^	140 × 10^−9^
PBS/light	198 ± 0.49	40	83 ± 0.9	14.4 ± 0.5	1.9 × 10^−6^	2 × 10^−6^
PBS/light EDTA	29.3 ± 1.4	72	12 ± 1	11.8 ± 2	288 × 10^−9^	1.6 × 10^−6^

#### PBS

From Table 3, it can be seen that the 4 w/v% substrate releases 13.7 ± 0.7 × 10^−10^ mol cm^−2^ min^−1^ of NO over a 24-hour period in PBS. As this measurement was obtained in the absence of light at room temperature, heat and light are not responsible for the decomposition of SNAP and subsequent NO release. It has been reported that PBS contains trace amounts of contaminating Cu, which catalyses the decomposition of SNAP. To confirm that this contributes to the release mechanism in our experiments we measured the NO release profile in PBS containing EDTA to chelate any contaminating Cu. With EDTA present the amount of NO released is reduced by 92% to 1 ± 0.32 × 10^−10^ mol cm^−2^ min^−1^ confirming that Cu was largely responsible for the NO release in PBS in the absence of light. When the same measurements are carried out in the presence of light without the chelator EDTA the amount of NO released over 24 hours 14.4 ± 0.5 × 10^−10^ mol cm^−2^ min^−1^. Upon the inclusion of EDTA, the amount of NO released over the 24-hour period is 11.8 ± 2 × 10^−10^ mol cm^−2^ min^−1^. As the amount of NO released in the presence of light, with or without metal chelators is similar, it suggests that SNAP decomposition in PBS is catalysed by both light and metal ions.

#### DMEM

In DMEM in the absence of light, the amount of NO released over a 24-hour period was 4.2 ± 1.5 × 10^−10^ mol cm^−2^ min^−1^ which is 69% lower than in PBS (Table 3). As discussed previously (section 3.1), DMEM contains a high concentration of glucose and both tryptophan and riboflavin that have also been shown to scavenge NO which can explain the lower concentrations of NO measured in this solution when compared to PBS. However, to determine if any Cu was also present in this solution, we also measured the release profile with additional EDTA. The amount of NO released in this solution was 3.4 ± 2 × 10^−10^ mol cm^−2^ min^−1^, not dissimilar to that measured without EDTA, suggesting that the EDTA was having little effect on Cu content in this solution. Along with tryptophan, DMEM also contains many other amino acids that have been shown to bind Cu. Sass-Kortsak *et al.* conducted a comprehensive study on the formation of amino acid–Cu complexes in serum and found that glutamine, threonine, cystine and most notably histidine bound to Cu. Furthermore, they also noted that certain combinations of amino acids increased the binding capability, histidine being the key amino acid.^40^ Therefore, in our experiments we can assume that the presence of these amino acids also lowers the NO payload in comparison to PBS by reducing the availability of Cu. There are no reports of the effects of alkaline pH on stability of SNAP specifically, but Hornyák *et al.* studied the decomposition of a similar RSNO, *S*-nitroso-*N*-acetylcysteine (SNAC) and although this occurred more slowly in basic conditions (pH 8.4–8.8), it was subject to decomposition.^41^ In the presence of light we observed only a 20% increase in the amount of NO released (5.3 ± 0.46 × 10^−10^ mol cm^−2^ min^−1)^ compared to the measurement in the dark and we also observed negligible reductions of NO payload in DMEM in the presence/absence of EDTA, or in the presence/absence of light respectively. This is in agreement with Sunda *et al.* who observed an increase in the dissociation constant of metal-EDTA complexes at high pH (7.7–9).^42^ As the pH of DMEM upon NO release increases up to a maximum of pH 9, this could cause the dissociation of any formed Cu-EDTA complexes increasing the availability of Cu and this may account for the lowest % reduction of NO payload in this solution.

#### LB broth

In the absence of light, the lowest payload of NO, over 24-hours, was observed in LB broth 0.59 ± 0.51 × 10^−10^ mol cm^−2^ min^−1^. As mentioned above (section 3.2) The LB broth used in these experiments contained B vitamins (B6 and 12), tryptophan and riboflavin (vitamin B2) which scavenge NO. As with the other solutions, the presence of Cu in this solution was confirmed by measuring the NO release profile in LB with EDTA. The concentration of NO released decreased by 98% to 0.1 ± 0.05 × 10^−10^ mol cm^−2^ min^−1^, confirming the chelation of Cu in the solution by EDTA. LB broth also contains amino acids known to bind Cu, however the concentration of these in this solution is much higher than in DMEM. For example, LB broth contains 10× more histidine than DMEM. A higher concentration of amino acids, particularly those that are known to bind Cu explains why the payload of NO released in LB is lower than in DMEM. In the presence of light (W/O EDTA) the amount of NO released over 24 hours increases by 92% (compared to the measured NO in the dark), 7.5 ± 0.03 to ×10^−10^ mol cm^−2^ min^−1^ and the payload is higher than that measured in DMEM. This suggests that breakdown of SNAP in this solution is catalysed predominately by light. However when EDTA is added to the solution any available Cu is chelated more efficiently (in comparison to DMEM), which explains the higher % reduction, as pH is significantly lower in LB compared to DMEM.

### 3.6 Antibacterial efficacy in nutrient rich conditions

**Fig. 5:**
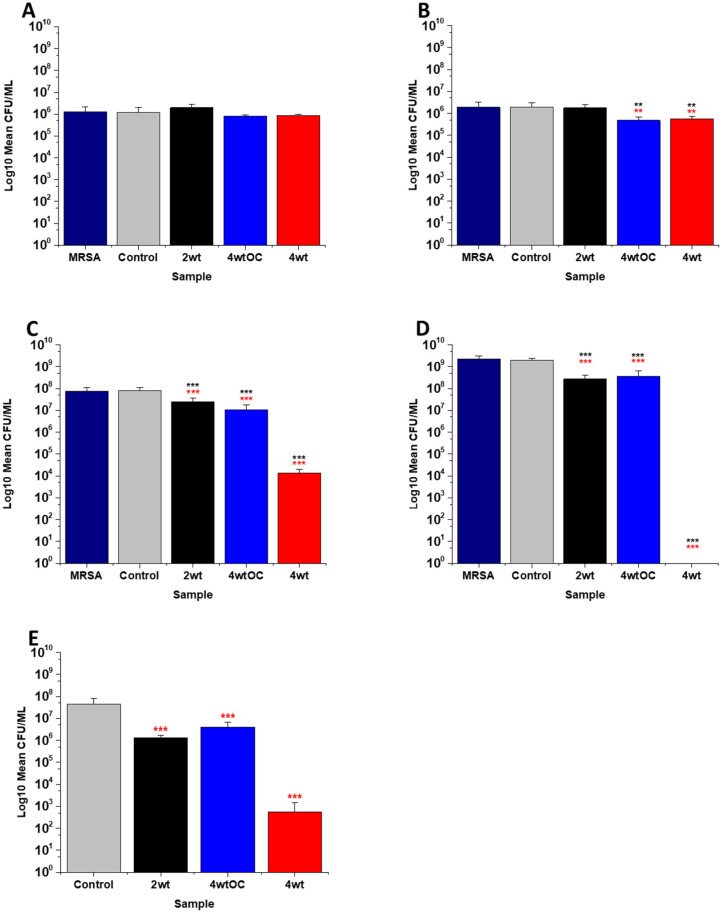
Antibacterial efficacy of NO releasing polymer surfaces in planktonic assays against MRSA under nutrient rich conditions (LB) after 1 (A), 2 (B), 4 (C), and 24 hours (D) incubation. Graph E shows the results obtained with NO releasing polymer surfaces in a 24 hours biofilm assay with MRSA under nutrient rich conditions. Values represent mean ± SD (*n* = 3; ***P ≤ 0.001; ** *P* ≤ 0.01; *P ≤ 0.05). Red represent SD from polymer control coated samples, black from bacterial control only.

**Fig. 6:**
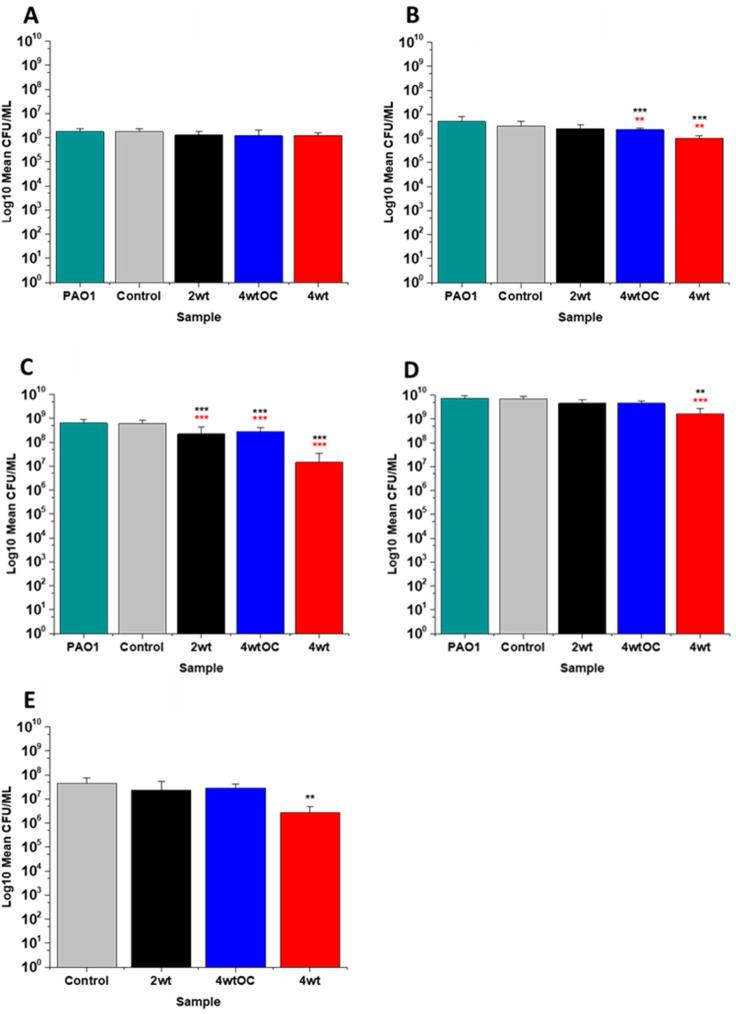
Antibacterial efficacy of NO releasing polymer surfaces in planktonic assays against PAO1 under nutrient rich conditions (LB) after 1 (A), 2 (B), 4 (C), and 24 hours (D) incubation. Graph E shows the results obtained with NO releasing polymer surfaces in a 24-hour biofilm assay with MRSA under nutrient rich conditions. Values represent mean ± SD (*n* = 3; ***P ≤ 0.001; ** *P* ≤ 0.01; *P ≤ 0.05). Red represents SD from polymer control coated samples, black from bacterial control only.

The antibacterial efficacy of the various SNAP loaded substrates was evaluated by planktonic assay with both Gram positive, MRSA (Fig. 5) and Gram negative, PAO1 (Fig. 6) bacteria in nutrient rich conditions (in LB broth) over a time period of 1, 2, 4 and 24 h. In experiments with MRSA, we observed that as time progresses, antibacterial efficacy increases and this follows the [NO] released (Table 2). After 1 hour (Fig. 5A) there was no reduction in the bacterial counts. After 2 hours (Fig. 5B), we began to see activity with the 4 w/v%/OC and 4 w/v% sample after a 2-hour incubation (53% and 73% respectively). At 4 hours (Fig. 5C) a 61%, 85% and 4 log reduction was observed for the 2 w/v%, 4 w/v%/OC and 4 w/v% substrates, respectively. At the 24-hour time point (Fig. 5D), a reduction of 82%, 89% and 100% was observed of the 2 w/v%, 4 w/v% /OC and 4 w/v% substrates, respectively. Increasing the total amount of SNAP in the substrate, increased the total payload of NO and consequently antibacterial efficacy (4 w/v% > 4 w/v%/OC > 2 w/v%) increased. The results also demonstrated that the addition of a polymer overcoat reduced the antibacterial efficacy of the substrate, most likely due lower NO release due to reduced water uptake into the hydrophobic overcoat. Alongside planktonic assays, we also determined the ability of the substrates to resist biofilm formation over a period of 24 hours in nutrient rich conditions as shown in Fig. 5E. We observed a 1 log reduction with 2 w/v % and 4 w/v %/OC and over 4 log reduction with 4 w/v%.

In the experiments with Gram negative PAO1 (Fig. 6), we observed a reduced antibacterial efficacy in comparison to MRSA. No antibacterial activity was observed with 2 w/v% samples at 1 (Fig. 6A) or 2 hours (Fig. 6B). At 4 hours (Fig. 6C), we observed a 62% reduction at 4 hours but there was no statistically significant reduction at 24 hours (Fig. 6D) as bacterial regrowth was observed. For the 4 w/v%/OC sample, no statistically significant reduction was observed at 1 hour, 42% was observed at both 2 (Fig. 6B) and 4 (Fig. 6C) hours and no statistically significant reduction was observed at 24 hours (Fig. 6D). Under nutrient rich conditions, the concentration of NO released from 2 w/v% or 4 w/v%/OC samples is not sufficient to sustain the antibacterial effects against PAO1. For the highest concentration of SNAP, the 4 w/v% sample, we observed a 41% reduction at 1 hour (Fig. 6A). This increased to 88% at 2 hours (Fig. 6B), 2 log reduction at 4 hours (Fig. 6C) but after 24 hours, the antimicrobial efficacy decreased to 82%. In biofilm assays with PAO1, we observed a 53% reduction with both 2 w/v% and 4 w/v%/OC samples and an 87% reduction with the 4 w/v% sample (Fig. 6E).

In our work antibacterial efficacy against Gram negative PAO1 is reduced in comparison to Gram positive MRSA and this is consistent with previous work reported by us.^43^ Our results are also in agreement with several other groups who have also observed reduced efficacy of SNAP functionalised materials against Gram negative bacteria.^44–49^ It is possible that PAO1 is more resistant to NO due to the presence of an alginate slime matrix coating. Simpson *et al.* found that the alginate coating allowed *P. aeruginosa* to scavenge reactive oxygen/nitrogen species and protected the bacterial cells from damage from these radicals.^50^ As NO activity is based on combining with radicals to form reactive nitrosative species that damage bacteria, and these can be scavenged by this slime layer, a much larger concentration will be required to elicit antimicrobial efficacy. Friedman *et al.* have also observed lower antibacterial efficacy with NO against Gram negative species including *Pseudomonas* and have attributed this to the presence of flavohemoglobin enzymes that neutralise NO.^51^ Both of these effects could explain the lowered antibacterial efficacy of our materials to PAO1. Additionally, the reduced activity may also be a consequence of nutrient rich media with an excess of scavengers in comparison to nutrient poor which will also affect the efficacy.

We acknowledge that there have been conflicting reports on the antibacterial efficacy of NO releasing materials against Gram positive *versus* Gram negative bacteria. For example, Schoenfisch *et al.* reported increased efficacy against Gram negative *P. aeruginosa vs*. Gram positive *S. aureus* and MRSA in separate comprehensive studies with NO donor modified chitosan and dendrimers.^52,53^ However, as both chitosan and the dendrimers used in Schoenfisch's study exhibit inherent antimicrobial activity, and the NO donor or release profile/payload differs it may account for these differences. Additionally, specific interactions between the functionalised material and the bacteria may also play an integral role, indeed Schoenfisch found different functional groups on chitosan or changes to the dendrimer size could influence the minimal bactericidal concentration (MBC) due to the interaction of the material with the bacterial cell membrane.^52^ In earlier work, the same group observed the reverse, with reduced *S. aureus* adhesion *vs*. *P. aeruginosa* in biofilm assays with NO releasing xerogel coated steel indicating that this material demonstrated an improved response against Gram positive bacteria.^54^ We conclude that bacterial response to NO is complex and variable dependent on the functionalised material and experimental conditions and this may account for the differences in the observations between research groups.

### 3.7 Antibacterial efficacy in nutrient poor conditions

**Fig. 7:**
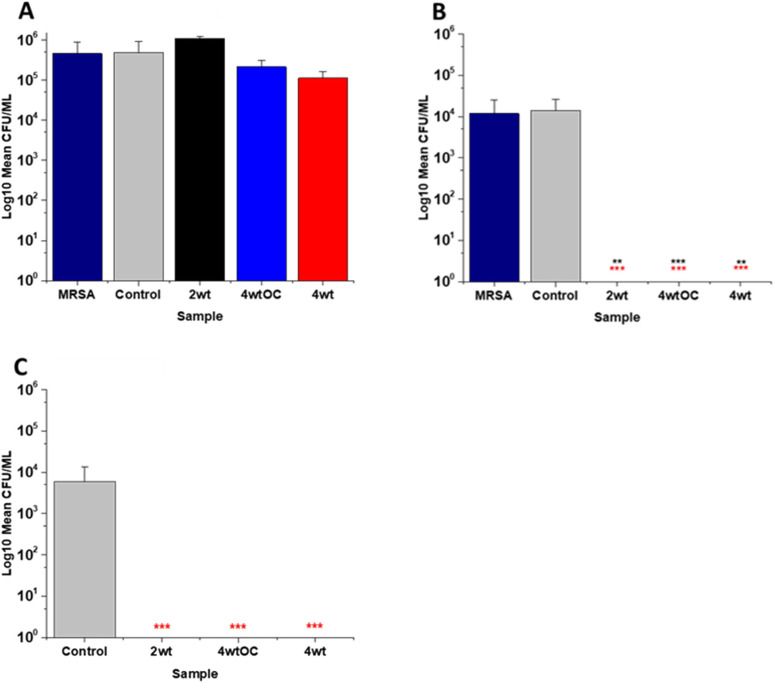
Antibacterial efficacy of NO releasing polymer surfaces in planktonic assays against MRSA under nutrient poor conditions (PBS) after 4 (A) and 24 hours (B) incubation. Graph C shows the results obtained with NO releasing polymer surfaces in a 24-hour biofilm assay with MRSA under nutrient poor conditions. Values represent mean ± SD (*n* = 3; ***P ≤ 0.001; ** *P* ≤ 0.01; *P ≤ 0.05). Red represent SD from polymer control coated samples, black from bacterial control only.

**Fig. 8:**
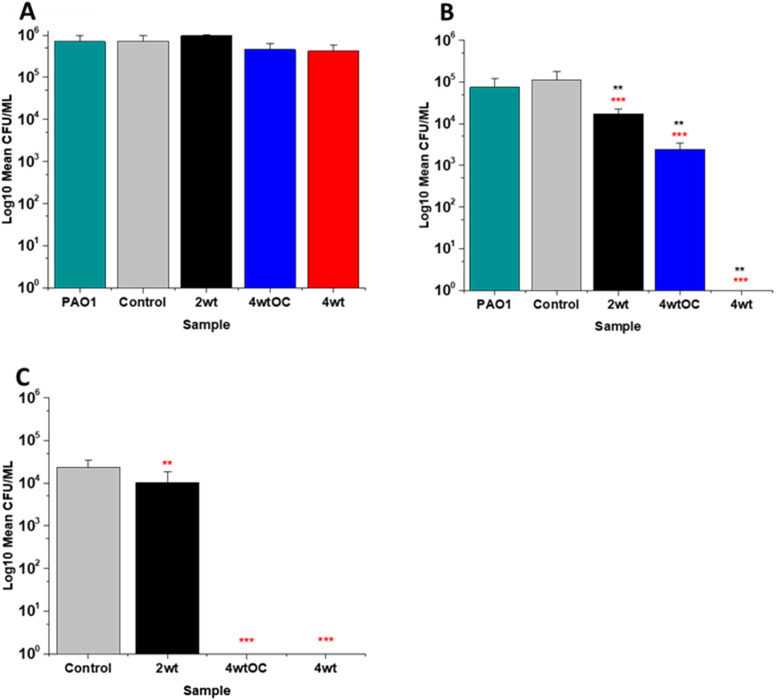
Antibacterial efficacy of NO releasing polymer surfaces in planktonic assays against PAO1 under nutrient poor conditions (PBS) after 4 (A) and 24 hours (B) incubation. Graph C shows the results obtained with NO releasing polymer surfaces in a 24-hour biofilm assay with PAO1 under nutrient poor conditions. Values represent mean ± SD (*n* = 3; ***P ≤ 0.001; ** *P* ≤ 0.01; *P ≤ 0.05). Red represent SD from polymer control coated samples, black from bacterial control only.

The antibacterial efficacy of the samples against MRSA (Fig. 7) and PAO1 (Fig. 8) was also determined in nutrient poor conditions (PBS). In the planktonic assay against MRSA, no reduction in number of bacteria were observed at 1 or 2 hours (ESI Fig. S5†) and no statistically significant reduction was observed at 4 hours (Fig. 7A). At 24 hours (Fig. 7B), a 100% reduction was observed with all substrates (2 w/v%, 4 w/v%OC and 4 w/v%). The ability of the substrates to prevent biofilm formation was evaluated after 24 h. All of the substrates (2 w/v%, 4 w/v%/OC and 4 w/v%) completely (100%) prevented MRSA biofilm formation and adhesion (Fig. 7C). The increased efficacy against biofilms observed in nutrient poor experiments may be due to the lack of nutrients available to support bacterial survival or the increased NO payloads in PBS.

In the planktonic assay against PAO1 there was also no antibacterial activity at 1 or 2 hours (Fig. S5 ESI†) and no statistically significant reduction was observed at 4 hours (Fig. 8A). At 24 hours (Fig. 8B), a 1 log reduction was observed for the 2 w/v% samples and a 100% reduction observed for 4 w/v% OC and 4 wt% samples respectively. In biofilm assays (Fig. 8C), we observed a 56% reduction in adhesion on the 2 w/v% surface whilst 4 w/v%/OC and 4 w/v% NO releasing polymer surfaces were 100% resistant to PAO1 adhesion.

The antibacterial efficacy results at the 1, 2- and 4-hour time points for both bacterial species were unexpected as we observed the highest payloads of NO released in PBS (Table 2). This was particularly surprising given the amount of NO released is likely to be higher in antibacterial efficacy tests as the samples are incubated at 37 °C. Indeed, Brisbois *et al*. conducted a comprehensive study of SNAP doped into medical polymers and found that NO release was around 30% higher (in the absence of light) when samples were incubated at 37 °C compared to room temperature due to thermal decomposition of the SNAP.^24^ Given the high payloads of NO we observed, and the absence of scavengers in PBS, we assume that there is another reason for the reduced antibacterial efficacy.

Time course pH measurements obtained in the different solutions (LB, DMEM and PBS) at 37 °C (Fig. 4) indicate that the pH of PBS in the first 4 hours ranges from 7.3 to 6.9 across all different samples. Whereas the pH in LB is much lower and ranges from 6.4 to 5.8. Peroxynitrite, a product of the reaction between NO and superoxide and a major contributor to antimicrobial activity, exists in different forms depending on the pH environment.^55^ At a pH of 7.4, 80% of peroxynitrite is in the anionic form ONOO^−^ (oxoperoxonitrate), whereas at lower pH (6.2), 80% exists in the protonated form, ONO_2_H (hydrogen oxoperoxonitrate).^56^ Although shortlived, the protonated form decays by homolysis of its peroxo bond to make highly antimicrobial OH and NO_2_ radicals.^57^ Whilst both forms of peroxynitrite are known to elicit antimicrobial effects it is possible that the depleted antimicrobial activity of the NO releasing substrates at the earlier timepoints in nutrient poor conditions are due to the peroxynitrite being present largely in its anionic form. This theory is in agreement with Hurst *et al.* whom conducted a comprehensive study to determine the toxicity of various peroxynitrite generated species against *E. coli* and concluded that ONO_2_H was the most toxic and bactericidal form of peroxynitrite.^58^ Similarly in a study by Iwaki *et al.*, ONO_2_H was found to have the most significant bactericidal/fungicidal effect on *S. mutans* and *C. albicans*.^59^

Along with the increased antibacterial effect of the protonated form, it has been shown that the anionic form of peroxynitrite can interact with Cu. Ghahramani *et al.* modified a chaperone protein commonly found in the eye with peroxynitrite and found that its ability to sequester Cu ions increased after modification. This in turn had a protective effect on lenticular tissue as it prevented Cu catalysed ascorbic acid oxidation.^60^ This paper reports for the first time a time course study (in nutrient rich and nutrient poor conditions) which allows for the hypothesis of the antimicrobial mechanism proposed. In our work, if the anionic form of peroxynitrite formed under nutrient poor conditions was interacting with Cu, this may also contribute to the reduced antimicrobial efficacy observed. Over the 24-hour time period, the antimicrobial activity resumes, this could be due to a slight reduction in the pH over time, which results in an increase in the production of the protonated form of peroxynitrite or could be due to the anionic form exerting antimicrobial activity.

### 3.8 Antiviral activity assays

Several studies have highlighted the potential of NO donors as antivirals for medical applications as they have been shown to inhibit the replication of several DNA and RNA viruses, including coronaviruses. Early studies by Saura *et al.* determined the effects of NO donors on the replication of Coxsackievirus.^61^ They discovered that NO nitrosylated the cysteine residue present in the active site of a protease (3Cpro) which in turn inhibited protease activity that was essential for viral replication. In early studies with Severe acute respiratory syndrome (SARS) virus, Åkerström and Mirazimi found that NO reduced viral RNA production and also reduced palmitoylation of the spike (S) protein which subsequently affected the interaction between the S protein and its receptor ACE-2 leading to a loss in infectivity.^62^ Similarly, in a more recent study specifically with SARS-CoV-2, Akaberi *et al.* found SNAP halted SARS-CoV-2 replication, most notably after 2 hours exposure to the NO donor. They attributed this in part to a 65% reduction in protease (Mpro) activity, an enzyme which is an essential part of the virus life cycle.^62–64^

**Fig. 9:**
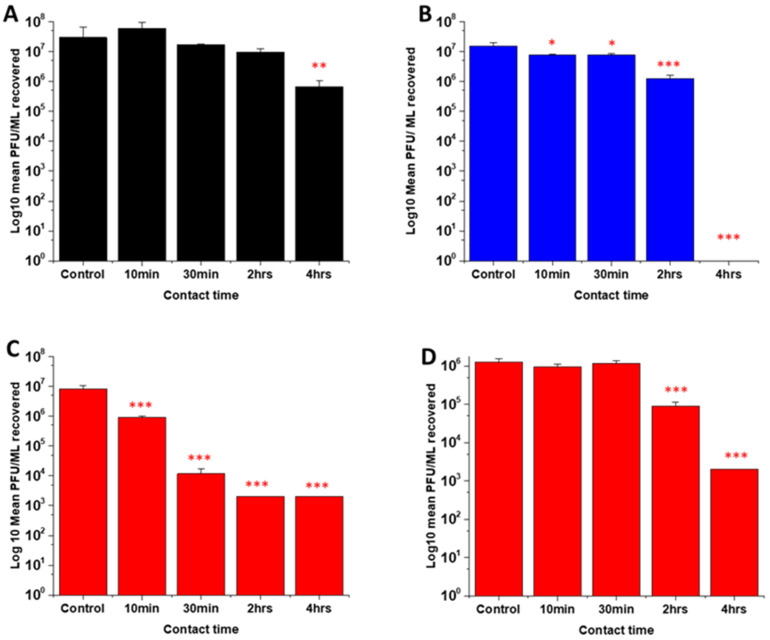
Average PFU mL^−1^ of SARS-CoV-2 virus recovered after contact with SNAP coated substrates for times ranging from 10 min to 4 h. Black = 2 w/v%; blue = 4 w/v%/OC; red = 4 w/v%. (A–C) Results obtained with Delta variant. Graph D shows average PFU mL^−1^ of Omicron variant recovered after contact with 4 wt% substrate. Values represent mean ± SD (*n* = 3; ***P ≤ 0.001; ** *P* ≤ 0.01; *P ≤ 0.05).

In this work, the antiviral activity of NO releasing substrates was determined by exposing substrates to the Delta (Fig. 9A–C) an Omicron variant of SARS-CoV-2 (Fig. 9D). A 1 log reduction (in comparison to control) was observed after 4 hours contact time with the substrate with lowest concentration (2 w/v%) of NO releasing polymer (Fig. 9A). When the concentration was doubled to 4 w/v% (Fig. 9C), 1 log reduction was observed after a shorter contact time (10 min), 3 log after 30 min and 4 log after 2 and 4 hours. The 4 w/v% OC substrates (Fig. 9B) displayed a 1 log reduction after 2 hours and >5 log reduction was observed after 4 hours, demonstrating that NO release and subsequent antiviral activity is slowed by the addition of the additional polymer layer. The findings from our studies with 4 w/v% coated substrates are in agreement with studies by Akaberi *et al.* who observed antiviral activity with NO after 2 hours.^63^

To determine if the substrates maintained antiviral activity against the Omicron variant of SARS CoV-2, the experiment was repeated with the 4 w/v% sample against the Omicron variant (Fig. 9D). A 1 log reduction was observed after 2 hours contact time and 4 log reduction was observed after 4 hours. Although we observed the same reduction (Omicron *vs*. Delta) at 4 hours, there is a reduction in the antiviral activity at shorter time periods. This may be due to the mutations between the variants. Omicron has 32 mutations in the spike protein and 15 mutations in the receptor binding domain and studies have demonstrated that it exhibits increased replication, particularly in certain cells compared to Delta.^50,65–68^ If these mutations result in an increased number of cysteine residues or increased protein residues available for palmitoylation, it is possible that higher concentrations of NO would be required to elicit the same antiviral effects at earlier timepoints and this warrants further investigation.

### 3.9 Cytocompatibility

**Fig. 10:**
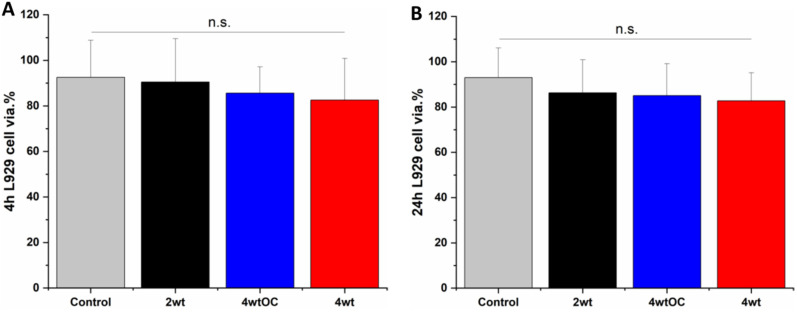
Cell viability of L929 cells after contact with leachate obtained after 4 hours (A) or 24 hours (B) incubation with SNAP coated substrates. Black = 2 w/v%; blue = 4 w/v%/OC; red = 4 w/v%. Results show over 70% cell viability demonstrating substrates are not cytotoxic.

Cell cytocompatibility experiments were performed on all surfaces using L929 mouse fibroblast cells as per ISO 10993 standard. The substrates were all submerged in DMEM media at 37 °C for 4 or 24 hours to allow leachates to diffuse into the media. The L929 cells were then challenged with this leachate containing media for 24 hours. We observed over 70% cell viability with all sample leachates, indicating that the substrates are not cytotoxic according to ISO 10993 standards (Fig. 10A and B).

## 4. Conclusion

The fabrication and functionalisation of nitric oxide (NO) releasing SNAP coatings for healthcare applications and a comprehensive study on the NO payloads observed in numerous relevant physiological conditions is reported. This is the first report of a time course study of the antimicrobial activity in both nutrient rich and nutrient poor conditions that allows us to hypothesise the mechanism of antimicrobial activity of SNAP Elast-Eon coatings. The coatings exhibit sustained and controlled payloads of NO and exhibit excellent antimicrobial activity against two serious resistant bacterial pathogens that are responsible for HCAI globally-Methicillin resistant *S. aureus* and *P*. *aeruginosa* and significant antiviral activity against two different variants of SARS-CoV-2 in short contact times. The coatings also demonstrated excellent cytocompatibility with over 70% cell viability observed after standard ISO testing. Due to the varying payloads of NO observed, this study also demonstrates the importance of determining NO release from functionalised materials under conditions relevant to the intended application. In conclusion, we have presented a simple method for the fabrication of coatings with a dual action antimicrobial/antiviral that could be used in healthcare settings and on PPE to mitigate transmission of pathogens commonly associated with HCAI. Furthermore, as SNAP-Elast-Eon is a versatile polymer that can be spin coated or dip coated to form homogeneous layers on many different materials, we hypothesise that it could also be applied to medical devices such as endotracheal tubes and nasal cannulas to prevent viral and bacterial co-infections that can lead to ventilator-associated pneumonia.

## Data availability

Data is available within the article or its ESI.† The data that support the findings is also available on request from the first author (Dr Jenny Aveyard zippy78@liverpool.ac.uk) and the corresponding author (Professor Raechelle D'Sa r.dsa@liverpool.ac.uk).

## Conflicts of interest

The authors declare no conflicts of interest.

## Supplementary Material

BM-012-D4BM00172A-s001
