# Supplementary material for: Nitric oxide releasing coatings for the prevention of viral and bacterial infections
Source: Biomater Sci. 2024 Jul 9;12(18):4664–81. doi: 10.1039/d4bm00172a (PMC11385708; doi:10.1039/d4bm00172a)
Supplement: Supplementary file 1 [file BM-012-D4BM00172A-s001.pdf]

Electronic Supplementary Material (ESI) for Biomaterials Science.
This journal is © The Royal Society of Chemistry 2024

Nitric Oxide Releasing Coatings for the Prevention of Viral and Bacterial Infections

Jenny Aveyard,[1] Siobhan Richards,[2] Man Li,[1] Graeme Pitt,[1] Grant L Hughes[2], Asangaedem Akpan[3,4] Riaz Akhtar,[1] Ahmed Kazaili[5] and Raechelle A D'Sa[1*]

[1.] School of Engineering, University of Liverpool, Harrison Hughes Building, Brownlow Hill, Liverpool, L69 3GH, UK

[2.] Departments of Vector Biology and Tropical Disease Biology, Centre for Neglected Tropical Disease, Liverpool School of Tropical Medicine, Pembroke Place, Liverpool, L3 5QA, UK

[3,] Department of Musculoskeletal & Ageing Sciences, University of Liverpool, Liverpool L69 3GL, UK,

[4.] Liverpool University Hospitals NHS FT, Liverpool L7 8XP, UK

[5]Department of Biochemistry & Systems Biology, University of Liverpool, Liverpool, L69 7ZB

Supplementary Information

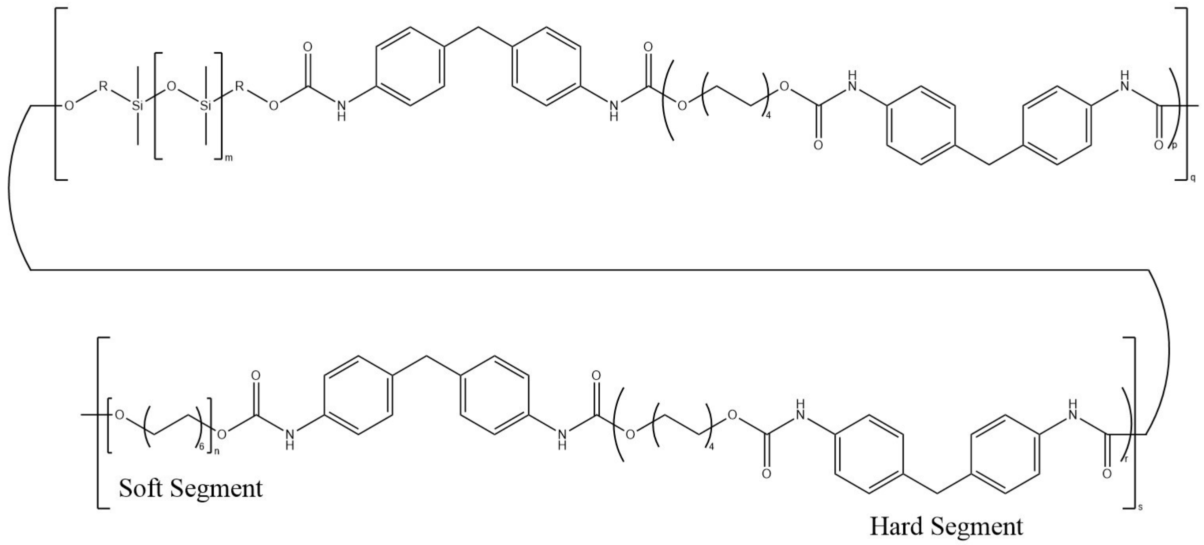

Figure S1: The structure of Elast-Eon type polymers. Elast-Eon is a urethane-silicone elastomer with a 40% hard segment content and a mixed polyether/siloxane soft segment. Structure taken from Gunatillake, P. A., Dandeniyage, L. S., Adhikari, R., Bown, M., Shanks, R., & Adhikari, B. (2019). Advancements in the Development of Biostable Polyurethanes. *Polymer Reviews*, *59*(3), 391–417. https://doi.org/10.1080/15583724.2018.1493694

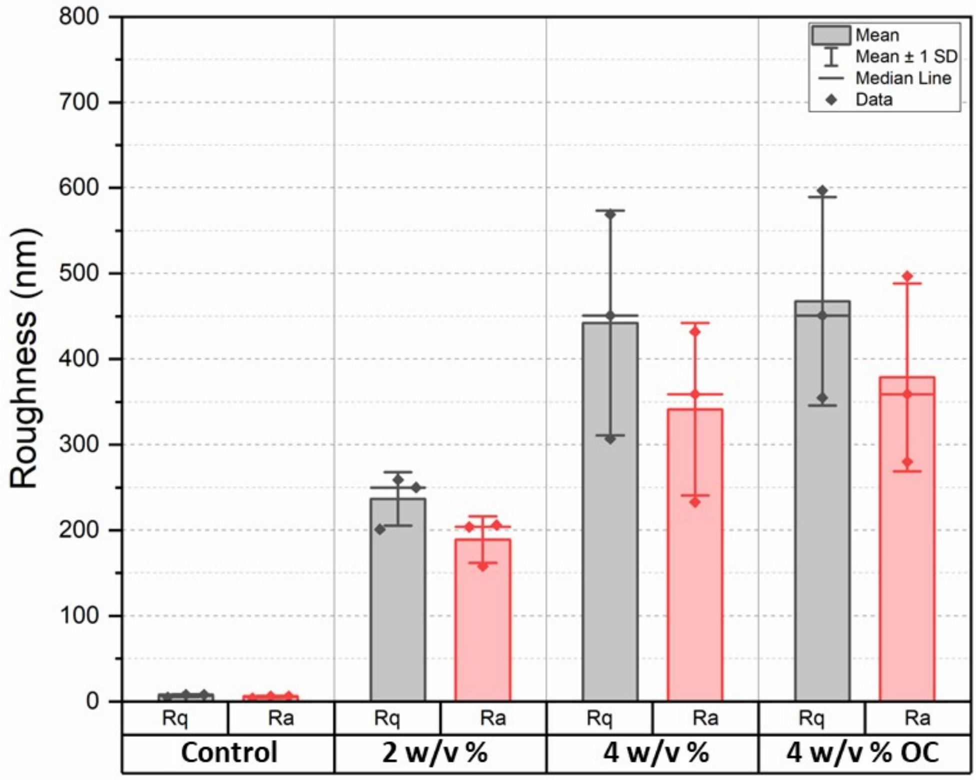

Figure S2: Graph detailing Rq/Ra results obtained from AFM measurement of SNAP coated substrates. As the concentration of SNAP increases, the roughness increases. The surface roughness of the substrates with a polymer overcoat is not significantly different to the 4 w/v% coated substrate, indicating that there is a thin layer of E2As applied to the surface.

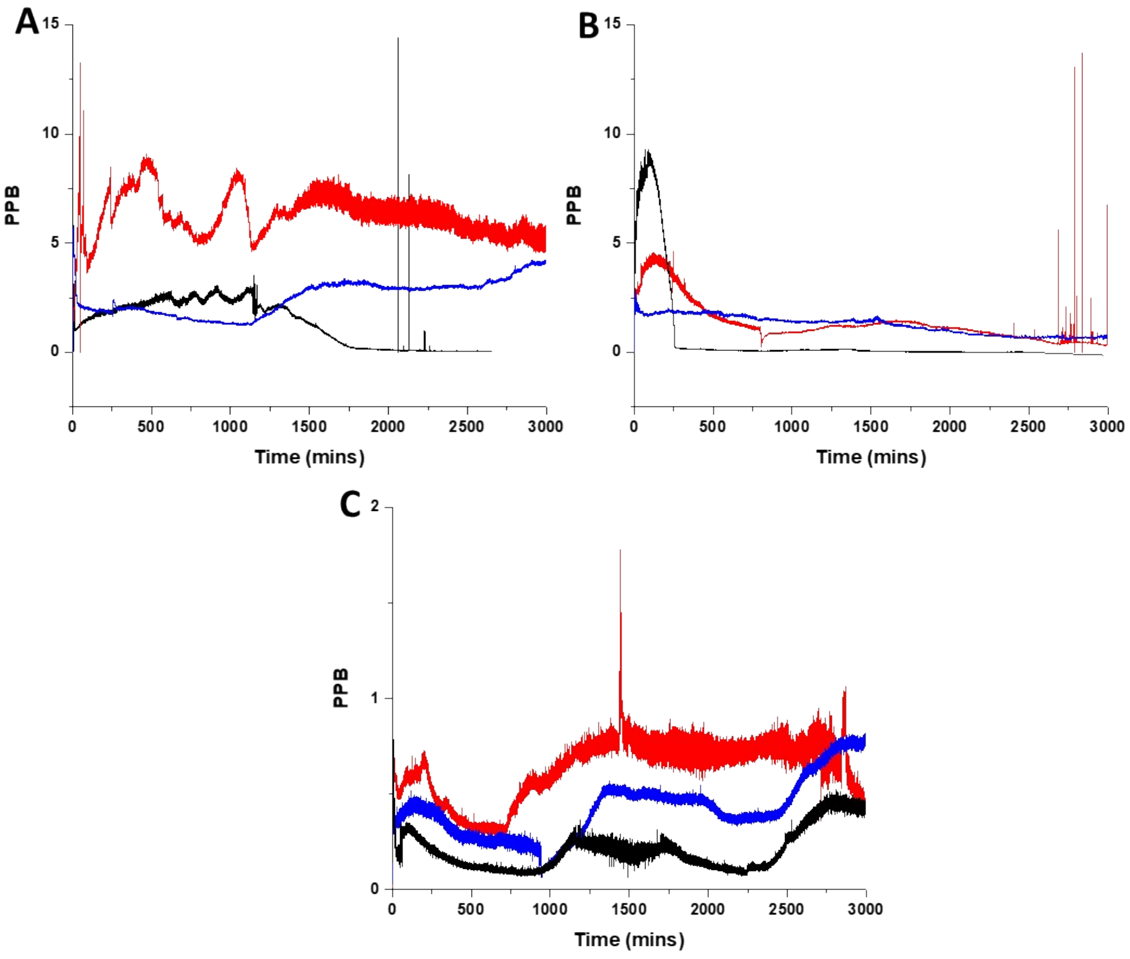

Figure S3: Representative chemiluminescence graphs of SNAP coated substrates in A) PBS B) DMEM C) LB Broth. Black= 2 w/v %; Blue = 4 w/v % OC; Red= 4 w/v %.

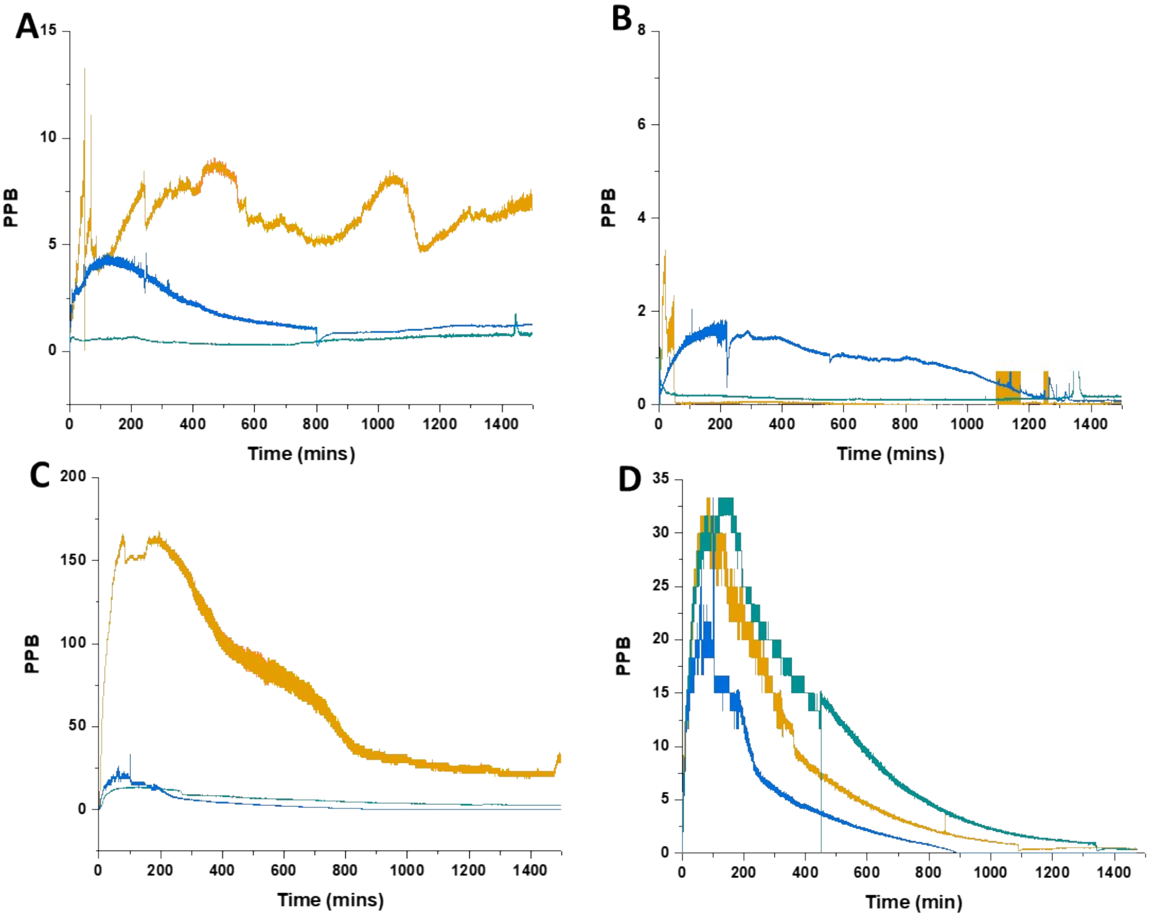

Figure S4: Representative chemiluminescence graphs of 4 w/v % SNAP coated substrates. A) Dark without EDTA B) Dark with EDTA C) Light without EDTA D) Light with EDTA. Orange= PBS; Blue = DMEM; Green= LB broth

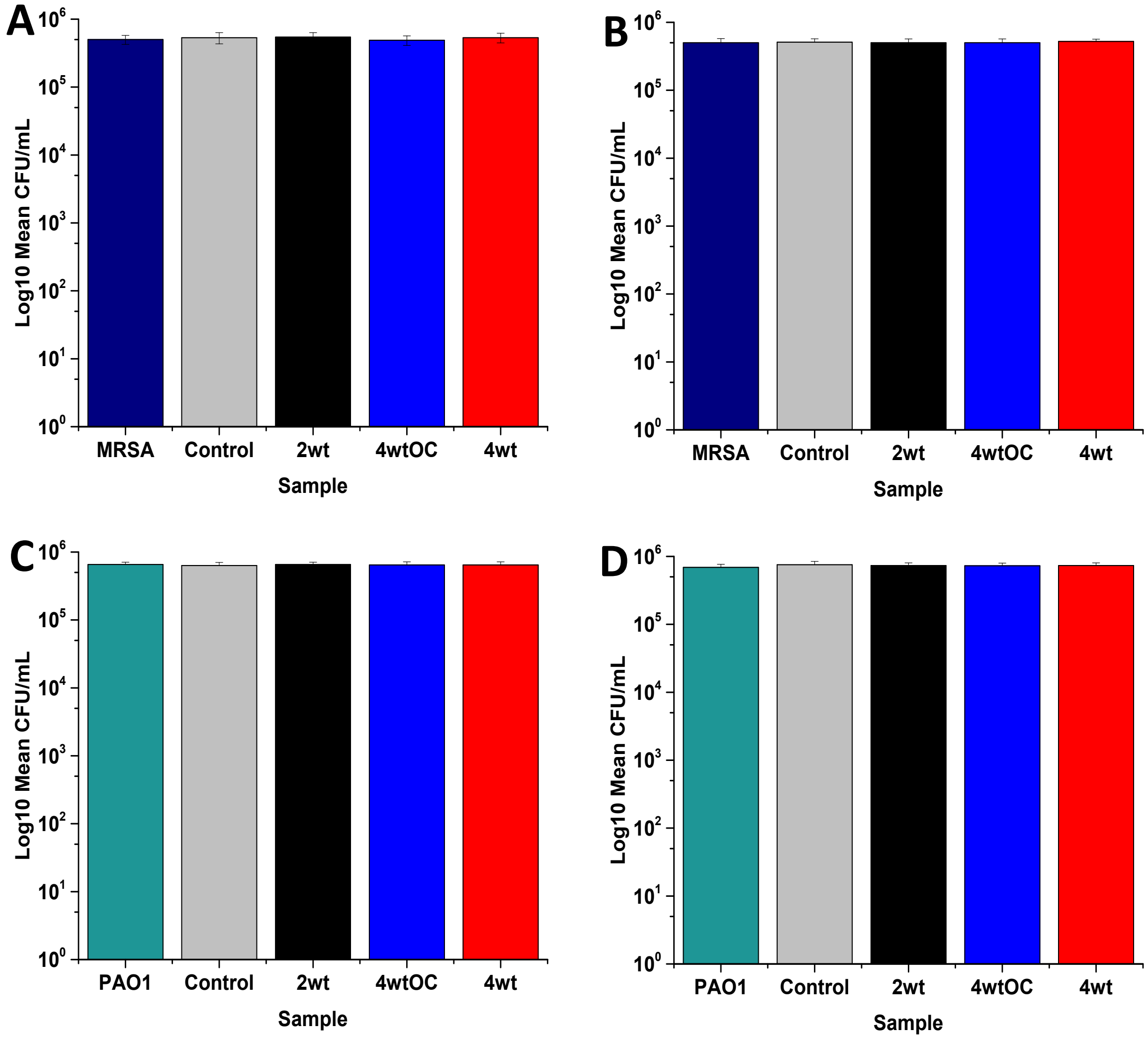

Figure S5: Antibacterial efficacy of NO releasing polymer surfaces in planktonic assays against MRSA (A and B) and PAO1 (C and D) under nutrient poor conditions (PBS) after 1 (A, C) and 2 hours (B, D) incubation. Values represent mean ± SD (n=3). There was no antibacterial activity observed with either bacteria at 1 or 2 hours with any samples tested.
